# Supplementary figures and images for: Nanoaperture fabrication via colloidal lithography for single molecule fluorescence analysis
Source: PLoS One. 2019 Oct 10;14(10):e0222964. doi: 10.1371/journal.pone.0222964 (PMC6786550; doi:10.1371/journal.pone.0222964)

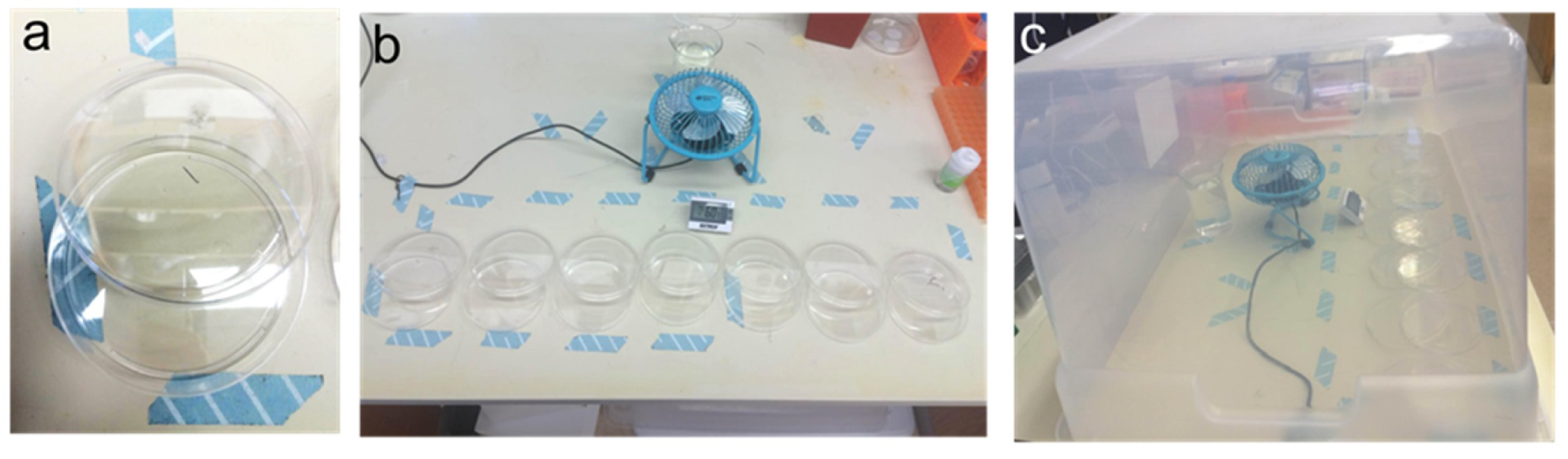

Supplement: S1 Fig — (a) Petri dish with lid partially ajar to exchange contents with humid air of the humidity chamber, in which cleaned coverslips are placed before being enclosed in the humidity chamber. (b) Layout of several petri dishes (containing coverslips), fan, beaker of water, and humidity monitor on the benchtop prior to covering with the “lid” of humidity chamber. (c) Humidity chamber enclosed by the “lid” consisting of an overturned plastic storage box. (TIF) [file pone.0222964.s001.tif]

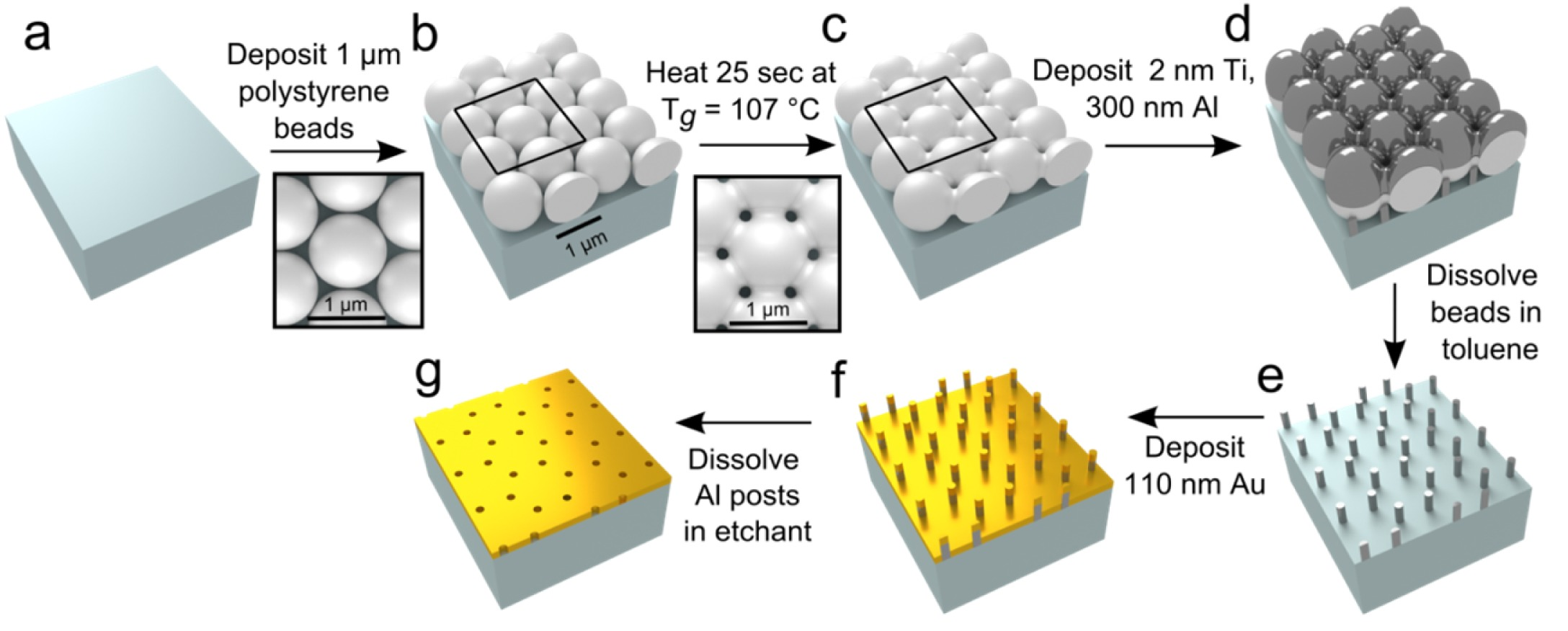

Supplement: S2 Fig — The procedure was similar to that for Al ZMWs shown in Fig 1 of the text. Black boxes represent expanded insets. Tg is the glass transition temperature of the polystyrene beads. For Au ZMWs aluminum was used as the post material. (TIF) [file pone.0222964.s002.tif]

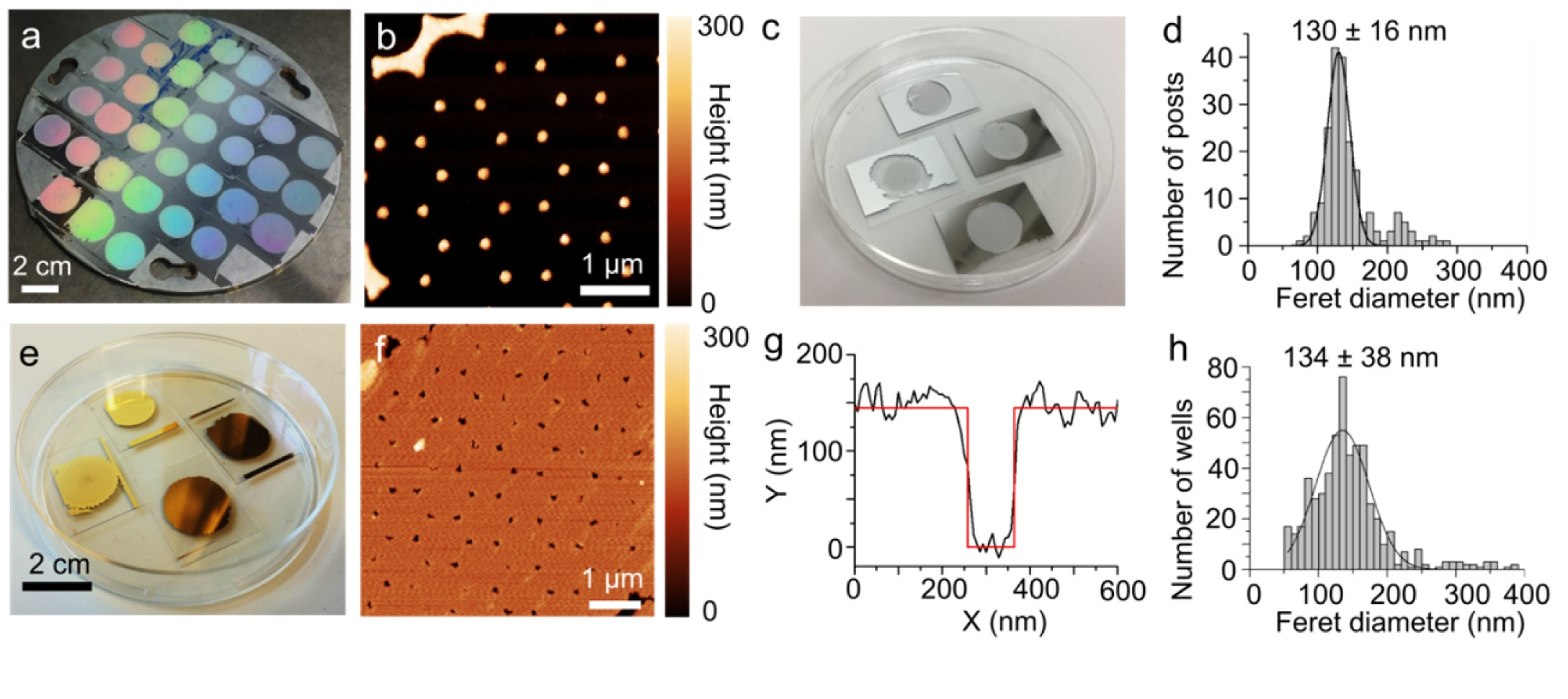

Supplement: S3 Fig — (a) Structural coloration from the diffraction of incident light following Al evaporative coating. (b) A representative AFM image of posts formed by Al deposition with the bead template annealed for 20 s. (c) Devices after Al deposition and bead lift-off. (d) Distribution of maxFeret diameters of posts measured at half-maximum height formed using a polystyrene bead template annealed for 20 s. (e) Completed gold ZMWs. (f) A representative AFM image of ZMW wells in the Au layer formed after dissolving the narrow Al posts. (g) AFM profile of an individual well. (h) Distribution of maxFeret diameters at half-depth of wells formed with a bead template that was annealed for 20 s. (TIF) [file pone.0222964.s003.tif]

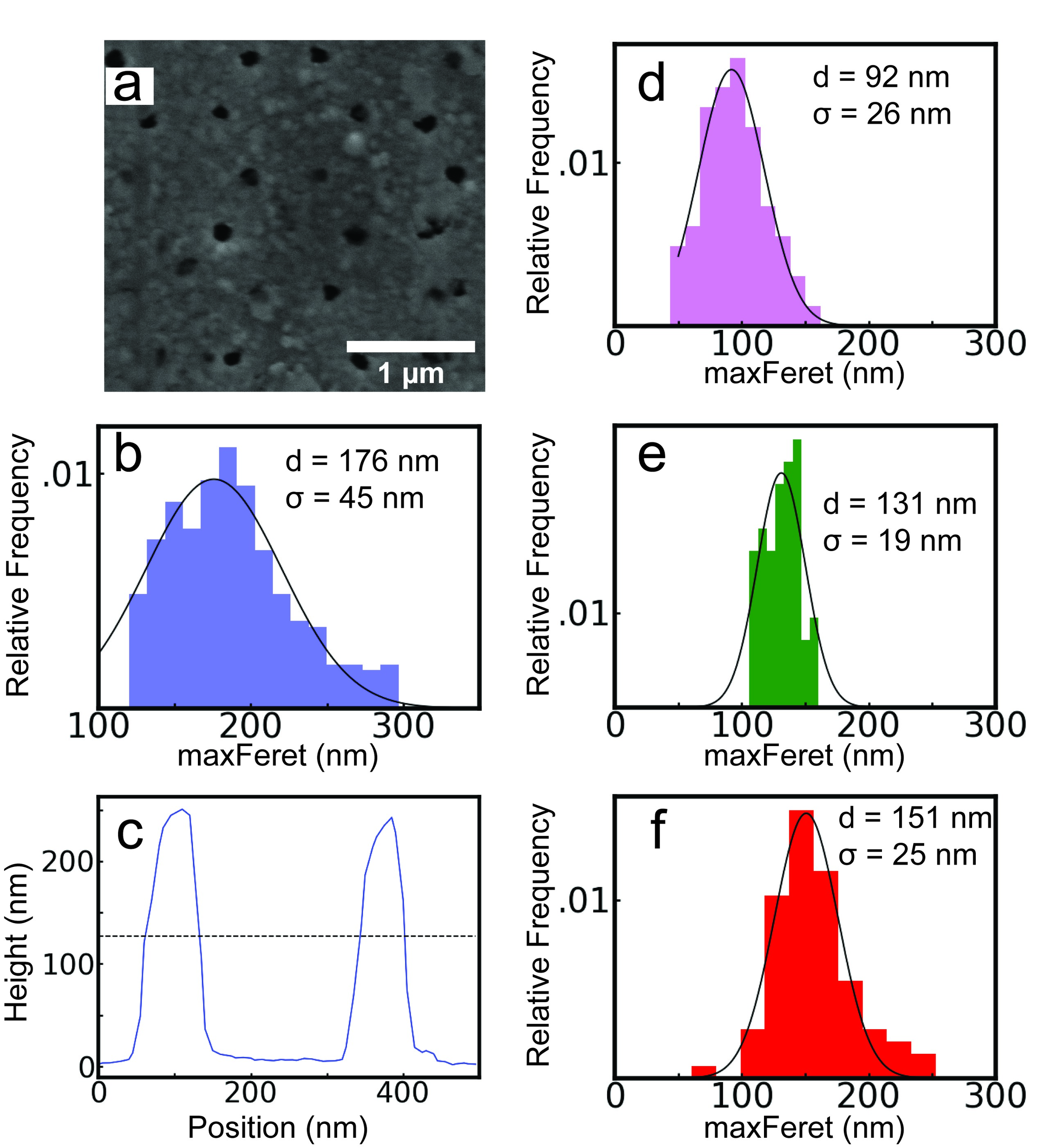

Supplement: S4 Fig — Differences in polystyrene bead annealing times lead to different pore sizes. (a) SEM image of a completed device. Pores are not perfectly cylindrical so they are characterized using maxFeret diameter. (b) Distribution of pore diameters from the device in (a) (bead annealing < 20 s) as characterized by SEM. (c) Cross-section of two posts after deposition. The dashed line represents the height to which metal is deposited in the next step. Note that the tapered post profile may cause a slight undercut in the final pores. (d)–(f) Distributions of pore diameters obtained from three different bead annealing times between 15 and 25 s. Mean maxFeret diameter at half-depth and standard deviations are listed. (TIF) [file pone.0222964.s004.tif]

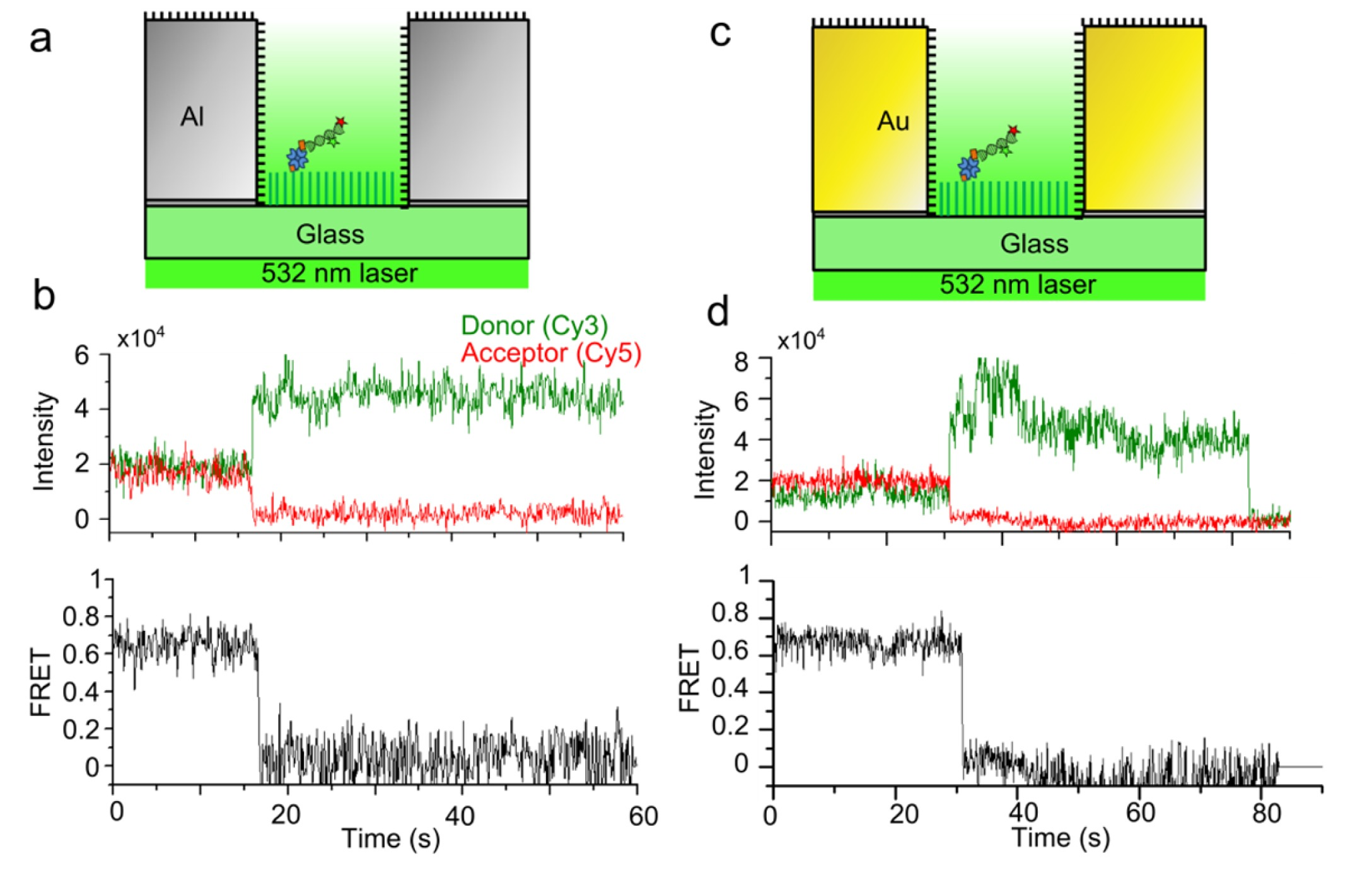

Supplement: S5 Fig — (A) Schematic cartoon of biotinylated Cy3-Cy5 dsDNA duplex immobilized in an Al ZMW while illuminated by 532 nm laser light. (B) Example smFRET trace of Cy3-Cy5 dsDNA duplex in an Al ZMW. (C) Schematic of biotinylated Cy3-Cy5 dsDNA duplex immobilized in an Au ZMW while illuminated by 532 nm laser light. (D) Example smFRET trace of Cy3-Cy5 dsDNA duplex in an Au ZMW. (TIF) [file pone.0222964.s005.tif]

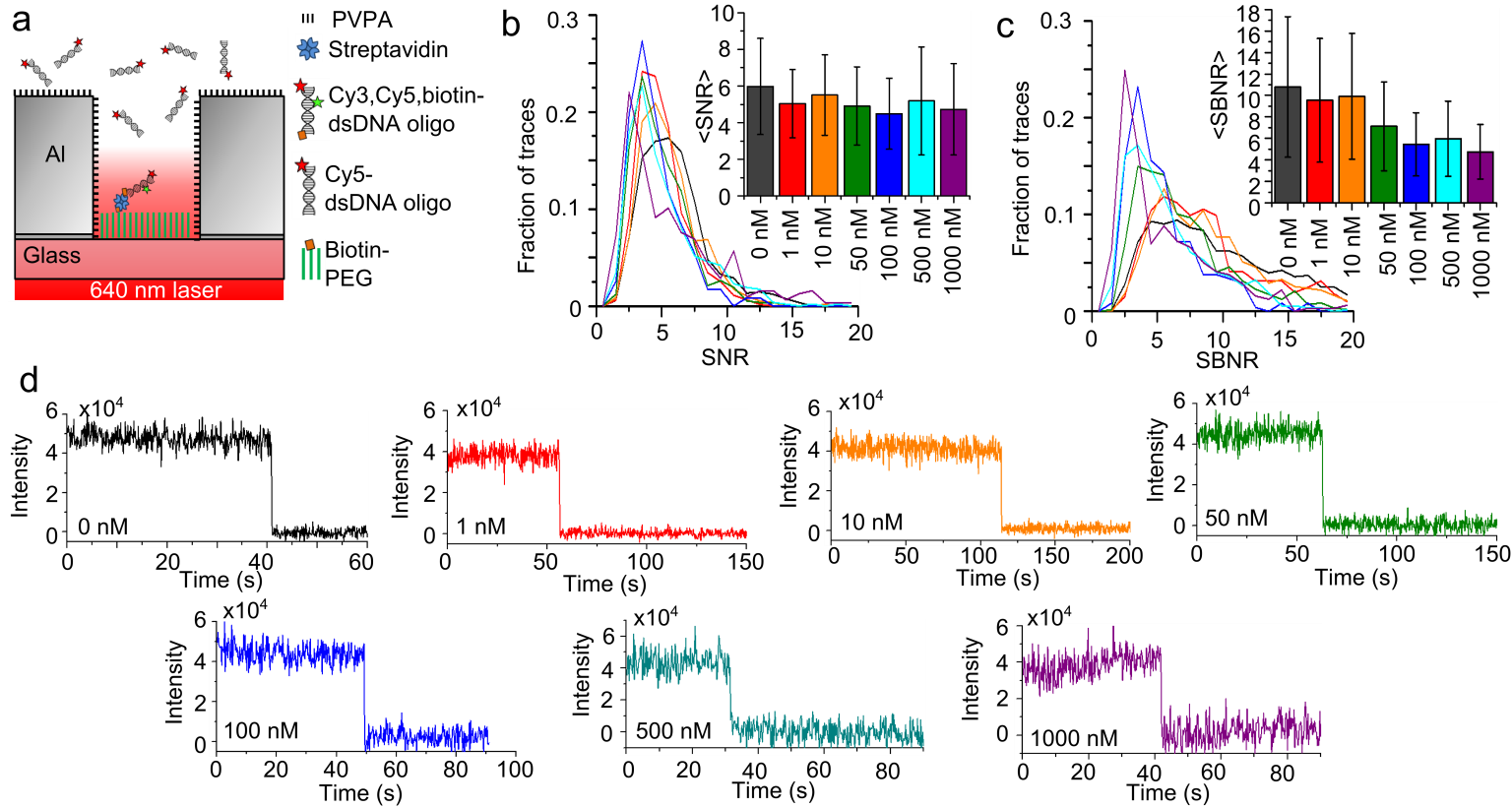

Supplement: S6 Fig — (A) Schematic cartoon of a biotinylated Cy3-Cy5 dsDNA duplex immobilized in an Al ZMW while illuminated by 640 nm laser light for direct excitation of Cy5, in the presence of non-biotinylated Cy5 dsDNA duplexes in the solution. (B) Line histograms depict the distributions of the SNR and (C) SBNR of Cy5 emission from immobilized DNA oligos under direct excitation at different concentrations of background Cy5 dsDNA, with bar charts indicating the mean ± s.d. (D) Example traces of Cy5 emission from direct excitation of immobilized oligos at 0 (n = 833), 1 nM (638), 10 nM (585), 50 nM (575), 100 nM (238), 500 nM (526), 1000 nM (286) concentrations. (TIF) [file pone.0222964.s006.tif]
